# Estimating actual COVID-19 case numbers using cumulative death count-A method of measuring effectiveness of lockdown of non-essential activities: a South African case study

**DOI:** 10.11604/pamj.supp.2020.35.2.24612

**Published:** 2020-07-01

**Authors:** Laura Cox, Clarence Suh Yah

**Affiliations:** 1 Wits Reproductive Health and HIV Institute (Wits RHI), Faculty of Health Sciences, University of the Witwatersrand, Johannesburg, South Africa; 2 School of Health Systems and Public Health, Faculty of Health Sciences, University of Pretoria, South Africa

**Keywords:** SARS CoV-2, infection fatality rate, testing coverage, case estimate

## Abstract

**Introduction:**

Estimating the number of SARS-CoV-2 infected individuals at any specific time point is always a challenge due to asymptomatic cases, the incubation period and testing delays. Here we use an empirical analysis of cumulative death count, transmission-to-death time lag, and infection fatality rate (IFR) to evaluate and estimate the actual cases at a specific time point as a strategy of tracking the spread of COVID-19.

**Methods:**

This method mainly uses death count, as COVID-19 related deaths are arguably more reliably reported than infection case numbers. Using an IFR estimate of 0.66%, we back-calculate the number of cases that would result in the cumulative number of deaths at a given time point in South Africa between 27 February and 14 April. We added the mean incubation period (6.4 days) and the onset-to-death time lag (17.8 days) to identify the estimated time lag between transmission and death (25 days, rounded up). We use the statistical programming language R to analyze the data and produce plots.

**Results:**

We estimate 28,182 cases as of 14 April, compared with 3,465 reported cases. Weekly growth rate of actual cases dropped immediately after lockdown implementation and has remained steady, measuring at 51.2% as of 14 April. The timing of drop in growth rate suggests that South Africa’s infection prevention strategy may have been effective at reducing viral transmission.

**Conclusion:**

Estimating the actual number of cases at a specific time point can support evidence-based policies to reduce and prevent the spread of COVID-19. Non-reported, asymptomatic, hard to reach and, mild cases are possible sources of outbreaks that could emerge after lockdown. Therefore, close monitoring, optimized screening strategy and prompt response to COVID-19 could help in stopping the spread of the virus.

## Introduction

Coronavirus disease-2019 (COVID-19), caused by the novel pathogen SARS CoV-2 (Severe Acute Respiratory Syndrome Coronavirus-2), is a respiratory illness that has infected at least 4 million people, and resulted in at least a quarter of a million deaths as of 9 May 2020 [1]. It has also resulted in enormous economic damage and social upheaval as many countries implement strict travel and movement restrictions, and so-called social distancing measures. There has been speculation that the world is experiencing a 1 in 100 year outbreak event, with similarities being noted between COVID-19 and the 1918 Spanish Flu [2]. First emerging in the Chinese province of Wuhan in late December 2019, SARS CoV-2 quickly spread to the rest of the world, earning it pandemic status [3]. While the number of new cases in China has dropped off dramatically, multiple new epicenters have emerged, including the USA, the UK, Italy, and Spain. The issue of symptom overlap with a multitude of other respiratory diseases, as well as growing evidence of asymptomatic transmission, makes this disease particularly sinister, and difficult to contain [4,5]. As there exists no vaccine or effective drug treatment [6], the focus has been on infection prevention strategies [7] as a means of slowing transmission rates and flattening the curve.

The COVID-19 crisis has presented the world with many new challenges. Amongst these is the issue of obtaining an accurate estimate of case numbers at a given point in time, in order to monitor the effectiveness of strategies aiming to curb transmission. Large scale testing has been advised by the World Health Organisation (WHO), as a method of obtaining estimates of case numbers to monitor the outbreak [8]. While testing widely is difficult for most countries, it is especially challenging for developing countries such as South Africa. South Africa recorded its first COVID-19 case on 5 March 2020. It began implementing increasingly strict infection prevention strategies that ultimately led to one of the world´s strictest nationwide lockdowns on 26 March. South Africa´s first death was recorded on 27 March [9]. Estimating the cumulative number of COVID-19 cases at any specific time point is difficult since large proportions of those infected will be asymptomatic or hard to reach, and will not be tested under most countries´ testing strategies. Studies on discrete populations have found the symptomatic ratio to be between 51.4% and 69% [10,11]. Relying purely on reported case numbers to assess the effectiveness of infection prevention strategies is not ideal, as it does not represent the true extent of infection spread. Additionally, case reports suffer from time lags due to the disease incubation period and testing delays, when unadjusted for these factors.

A method for empirically estimating actual number of cases coupled to a specific time point is needed to monitor effectiveness of infection prevention strategies such as nationwide lockdowns. Using cumulative death count may produce more reliable estimates of actual case numbers than relying on test results alone, as COVID-19 related deaths are arguably more reliably reported than infection numbers, both in magnitude and timing. We propose an empirical method for estimating true case numbers based on publicly available cumulative death count, best estimate infection fatality ratio (IFR), best estimate onset-to-death time lag, and best estimate mean incubation period. Alternative methods using cumulative death count to estimate actual case numbers have been proposed, such as Jombart et al. [12] model to estimate number of cases in a population experiencing newly reported COVID-19 related deaths. However, this model uses case fatality rate (CFR) as opposed to IFR. Here CFR is the proportion of confirmed cases that are fatal. Additionally, Jombart´s model uses a sophisticated method to estimate symptom onset date of cases calculated from death reports. Our method is distinguished from other work using death count to establish accurate case numbers as it does not rely on complex epidemiological models and is not intended to produce forecasts or projections. Additionally, our estimates of actual case numbers will be coupled with a point in time representing their transmission point, to allow analysis of interventions aimed at reducing transmission of the virus. Actual case numbers may be validated by serological surveys, once conducted.

## Methods

Data Collection: data on the number of COVID-19 cases and deaths (from 5 March to 9 May, 2020) were obtained from the South Africa´s National Institute for Communicable Diseases (NICD) website [9]. The data was analyzed using the statistical programming language R [13], and plots were produced using the package ggplot2 [14].

Data analysis: the estimated value for IFR (0.66%) was obtained from Verity et al. [15], as was an estimate for mean onset-to-death time lag (17.8 days). It should be noted that Verity et al. use date of positive test as a proxy for onset date. Mean incubation period was set at 6.4 days [16]. Mean transmission-to-death time lag was therefore set to 25 days (mean onset-to-death plus mean incubation period, rounded up). We use the following equation to describe the relationship between number of deaths, IFR, and transmission-to-death time lag. We use Dt to denote cumulative deaths at date t, and It-25 to denote actual number of infections (cases) at date t-25 days, and thus:

$$D_{t}=IFR\times I_{t-25}$$

$$I_{t-25}=\frac{D_{t}}{IFR}$$

In order to compare the actual case numbers with reported case numbers, we corrected reported case numbers for disease incubation period, by allocating them to date t-7 days (incubation period of 6.4 days rounded up), thus allowing comparison by point of transmission. Lastly, we calculated the rolling weekly growth rate (RWGR) of actual and reported cases (by point of transmission) as follows, where C denotes cumulative cases (actual or reported):

$$RWGR_{t}=\frac{C_{t}-C_{t-7}}{C_{t-7}}$$

## Results

**Figure 1:**
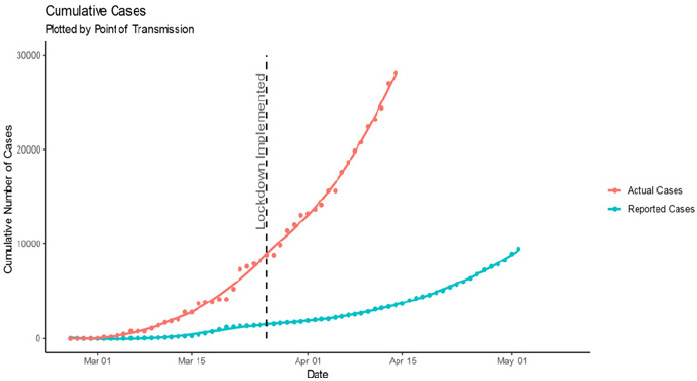
Cumulative actual cases versus cumulative reported cases over time

**Cumulative case numbers:** Figure 1 shows the cumulative actual vs cumulative reported cases over time, plotted by point of transmission. The vertical line shows the point in time where national lockdown was implemented. Note that actual cases can only be calculated up to 14 April due to the 25-day lag associated with case number calculation. We overlaid a LOESS (locally estimated scatterplot smoothing) line with an alpha of 0.5 for both data sets, to show local trends. We can see here that actual case numbers are much higher than reported case numbers. Figure 1 shows that South Africa has entered the exponential growth phase characteristic of many outbreaks and already seen around the world in the current COVID-19 outbreak. As expected, our estimate of cumulative actual cases is much larger than cumulative reported cases, with the difference between the two becoming greater over time. We estimate 28,182 cases as of 14 April, compared with 3,465 reported cases (adjusted for comparison by point of transmission).

**Figure 2:**
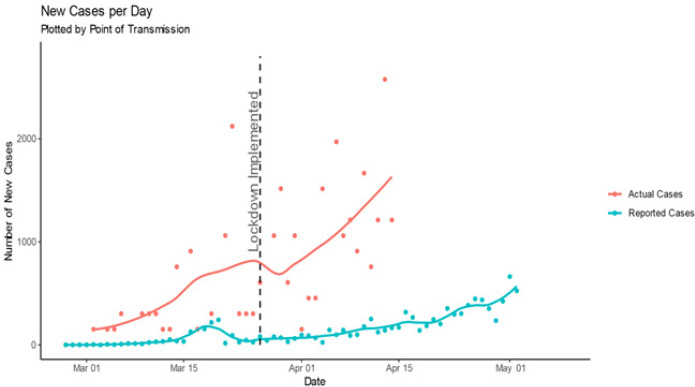
New actual cases per day versus new reported cases per day

**Figure 3:**
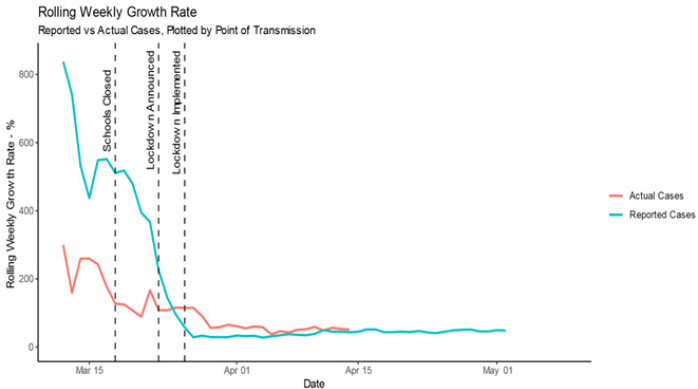
Rolling weekly growth rate of actual versus reported cases over time

**Growth rate:** we have assessed growth in case numbers in two ways: absolute new cases per day and RWGR. In an outbreak of a pathogen with an Rt > 1 (while the exact Rt of SARS CoV-2 is debated and potentially geographically variable, it is certainly greater than 1), we would expect the number of new cases per day to increase over time, leading up to the outbreak peak. Plotting new cases per day over time furnishes only a general overview of disease spread. Hence, we also calculate the RWGR, the number of new cases per week, as a proportion of cumulative number of cases up to the end of the previous week. We calculated this value on a rolling weekly basis, shifted by one day, to produce a smoothed weekly growth rate over time. Figure 2 depicts the number of new cases per day. We overlaid a LOESS line with an alpha of 0.6 for actual cases, and 0.2 for reported cases, to show local trends. Immediately post lockdown, the actual cases trend line drops sharply, indicating the average number of new actual cases per day dropped. The timing of this suggests that it may be as a result of lockdown implementation. This is in contrast to the trend line of new reported cases per day, which remains constant after lockdown implementation. This is consistent with the assumption that reported cases are not a true representation of infection numbers and remain subject to testing strategies and capacities. To assess growth rate, we calculated RWGR for actual and reported cases plotted in Figure 3. After a steadily decreasing (albeit fairly erratic) initial period, RWGR of reported cases converged on that of actual cases. As of 14 April, actual cases had a RWGR of 51.2%, and reported cases had a RWGR of 43.5%. In contrast, the RWGR of actual cases has remained steadier (due to the pattern of deaths) but experienced a decrease post lockdown implementation. This could suggest that the national lockdown has, to an extent, been successful at slowing viral transmission. The convergence of growth rate in actual and reported cases suggests that the growth in reported cases has become a more reasonable proxy for growth in actual cases over time. A fairly constant growth rate is consistent with exponential growth in cumulative case numbers as apparent in Figure 1. Actual case numbers are growing at approximately 50% per week.

**Figure 4:**
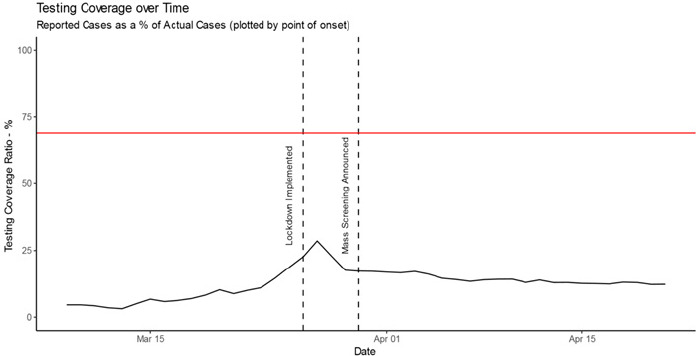
Testing coverage over time, with a horizontal line marking the 69% upper estimate symptomatic ratio of COVID-19

**Testing coverage:** Figure 4 shows the testing coverage over time. Here we represent testing coverage as cumulative number of reported cases as a proportion of actual cases. We plot this by point of onset, as this is the point at which an individual would present for testing if symptomatic. We would expect this to have an upper bound of 69% (the horizontal red line in Figure 4) to reflect the symptomatic ratio estimate [10,11]. Specifically, given that South Africa is employing a testing strategy that involves only testing symptomatic individuals, we would expect, at best, a testing coverage of 69%. Figure 4 plot shows that testing coverage steadily improved over time peaking at 28.6% on 27 March, but experiencing a drop immediately post lockdown implementation. This may be as a result of individuals being reluctant to leave their homes to seek testing as a result of uncertainty during the first few days of lockdown (despite the lockdown making explicit allowance for seeking medical services). After this drop, testing coverage remained fairly constant, measuring at 12.3% by 21 April. A reasonably constant testing coverage despite the exponential growth in case numbers suggests that the number of tests performed is growing to meet the increase in cases. This may be attributed to the introduction of a mass screening programme.

## Discussion

As of 9 May 2020, the COVID-19 outbreak has resulted in more than 250,000 deaths and has affected almost every country in the world [1]. There are currently 6 vaccines undergoing clinical evaluation, many more in pre-clinical stages [17], and many drug treatment trials underway. However, regulatory processes mean that these are most likely at least 18 months away. In the meantime, efforts to curb transmission of the virus are at the forefront of the fight against COVID-19. Monitoring the effectiveness of these efforts relies heavily on an accurate account of case numbers over time. Currently in most settings, testing strategies only target individuals showing symptoms. As such it is reasonable to assume that only a subset of cases is being captured, and the proportion this subset represents is not easily quantifiable. Additionally, reported case numbers suffer heavily from time lags due to testing delays, capacity issues, and the disease incubation period.

We propose a method of estimating case numbers using the more reliably reported cumulative death count along with estimated transmission-to-death time lag and IFR. Our results indicate that actual case numbers of COVID-19 are growing exponentially in South Africa. We estimate 28,182 cases as of 14 April, compared with 3,465 reported cases (adjusted for comparison by point of transmission). New cases per day dropped sharply post lockdown implementation; the timing of this is suggestive of lockdown measures slowing transmission. RWGR of actual cases also dropped post lockdown, which further adds to the suggestion that lockdown measures have slowed viral transmission. Post lockdown, actual case RWGR has remained fairly steady. A fairly constant growth rate is consistent with exponential growth in cumulative case numbers, as shown in the plot of cumulative case numbers. Additionally, the convergence of growth rate in actual and reported cases suggests that the growth in reported cases has become a more reasonable proxy for growth in actual cases over time. As a measure of how effective the country´s testing strategy is, testing coverage steadily improved from the beginning of the outbreak, and peaked at 28.6% on the 27 of March, but dropped immediately post lockdown (potentially as a result of lockdown). After this drop, testing coverage remained fairly constant, measuring at 12.3% on the 21 of April. A reasonably constant testing coverage despite the exponential growth in case numbers suggests that the number of tests performed is growing to meet the increase in cases. This may be attributed to the introduction of a mass screening, contact tracing, coverage, and general awareness and knowledge of COVID-19 [7].

Limitations estimating actual number of cases based on number of deaths recorded may be useful for retrospective monitoring of effectiveness of prevention strategies. However, a limitation is that the method relies mainly on best estimates to calculate case numbers and assign them to a specific point in time. Additionally, it cannot be used to infer mortality rate or infectivity of this disease, as it assumes these values. Lastly, it cannot be used in real time (due to the incorporated time-lag) or to produce projections or forecasts. Instead, it should be used to infer a general pattern of disease spread.

## Conclusion

Our results indicate that strict national lockdown of non-essential activities may slow the spread of COVID-19. Estimating the actual number of cases at a specific time point can provide support for evidence-based policies to prevent and reduce the spread of COVID-19. Effective lockdown of non-essential activities, quarantining, and isolation can cut off the route of transmission and reduce the overall size of the outbreak. Non-reported, asymptomatic, hard to reach and mild cases are possible sources of outbreaks that could emerge after lockdown. Therefore, close monitoring, optimized screening strategy and prompt response to COVID-19 cases could help in stopping the spread of the virus.

### What is known about this topic

- Using case fatality rate as an estimate of mortality of COVID-19 is problematic as it relies on positive tests results, which in turn relies on a country’s testing strategy. Additionally, COVID-19 case reports suffer from a time-lag due to testing delays and the disease’s inherent incubation period;
- Many individuals infected with SARS-CoV-2 will be asymptomatic, and remain untested, but nearly all of those who die from the infection will be recorded;
- Several epidemiological models have been developed to provide estimates of actual case numbers based on number of deaths, but these rely on case fatality rate.

### What this study adds

- This study proposes an empirical method for estimating actual case numbers of COVID-19 based on number of deaths, as a way of accommodating and accounting for asymptomatic cases;
- This method introduces a way to account for the time-lag due to the disease’s incubation period, to couple the estimate of number of cases to a point in time representing transmission;
- An estimate of actual case numbers closely coupled to a point in time representing transmission allows for analysis of interventions aimed at reducing transmission of the virus, and we apply this to the South African context.

## Competing interests

The authors declare no competing interests.
